# Rules regarding Marijuana and Its Use in Personal Residences: Findings from Marijuana Users and Nonusers Recruited through Social Media

**DOI:** 10.1155/2015/476017

**Published:** 2015-10-20

**Authors:** Carla J. Berg, David B. Buller, Gillian L. Schauer, Michael Windle, Erin Stratton, Michelle C. Kegler

**Affiliations:** ^1^Department of Behavioral Sciences and Health Education, Emory University School of Public Health, 1518 Clifton Road NE, Atlanta, GA 30322, USA; ^2^Klein Buendel, Inc., 1667 Cole Boulevard, Suite 225, Golden, CO 80401, USA; ^3^Immunization Services Division, National Center for Immunization and Respiratory Diseases, Centers for Disease Control and Prevention, 1600 Clifton Road NE, Atlanta, GA 30329, USA

## Abstract

Recent changes in policy and social norms related to marijuana use have increased its use and concern about how/where marijuana should be used. We aimed to characterize rules regarding marijuana and its use in homes. We recruited 1,567 US adults aged 18–34 years through Facebook advertisements to complete an online survey assessing marijuana use, social factors, perceptions of marijuana, and rules regarding marijuana and its use in the home, targeting tobacco and marijuana users to ensure the relevance of this topic. Overall, 648 (41.6%) were current marijuana users; 46.0% of participants reported that “marijuana of any type is not allowed in their home or on their property.” Of those allowing marijuana on their property, 6.4% prohibited *use* of marijuana in their home. Of the remainder, 29.2% prohibited *smoking* marijuana, and 11.0% prohibited *vaping, eating, or drinking* marijuana. Correlates of more restrictive rules included younger age, being female, having <Bachelor's degree, not having parents or people living with them who use marijuana, perceiving use to be less socially acceptable and more harmful, and being a nonuser (*p*'s <.05). Attitudes and subjective norms regarding marijuana are correlates of allowing marijuana in residential settings. Future work should examine areas of risk regarding household marijuana rules.

## 1. Introduction

Marijuana is the most commonly used illicit drug in the US, particularly among young adults, with marijuana use prevalence increasing in recent years [1]. Chronic marijuana use is associated with cognitive impairment and addiction [2]. While marijuana can be used in various forms (e.g., vaporized and edible), the most common mode of use is smoking [3], which is implicated in long-term effects on lung function [4–6], chronic bronchitis and respiratory irritation [2, 4], and impaired immunological competence of respiratory systems [7].

Marijuana smoke includes nitric oxide, nitrogen oxide, hydrogen cyanide, and aromatic amine (which is responsible for the mutagenic and carcinogenic activity of cigarette condensates) and includes more chemicals than tobacco smoke [8]. In addition, risk factors of adolescent marijuana use include peer influence, home environment, and parental monitoring [9]. Thus, marijuana use in the home may impact health of nonusers and increase the likelihood of youth initiation.

Recent efforts regarding legalization and decriminalization of marijuana use in the US [10] restricts use to personal residential environments. Thus, we must understand how marijuana use is being voluntarily regulated in these settings; moreover, it is critical to understand correlates of having fewer restrictions on marijuana use in these settings to potentially inform interventions aimed at increasing certain restrictions. The Theory of Planned Behavior (TPB) [11], with its focus on the impact of attitudes, subjective norms, and perceived behavioral control on behavior, has been applied to adopting home restrictions for cigarette smoking and thus may be an appropriate framework for this issue. Notably, research has shown that attitudes about marijuana use and expectations of family members and close friends (i.e., subjective norms) are influential in predicting the adoption of smoke-free rules in residential settings [12, 13].

Given the importance of this emerging and complex public health issue, our research aims were to (1) characterize rules regarding marijuana and its use in households among young adult population recruited via an online social networking site and (2) document correlates of having less restrictive rules including sociodemographic factors, personal marijuana use, social factors, and perceptions of marijuana risk.

## 2. Methods

### 2.1. Procedures

The Emory University Institutional Review Board approved this study, IRB# 00073636. We recruited participants aged 18–34 years via advertisements on Facebook, a social networking website, targeting tobacco and marijuana users and nonusers. Recruitment occurred over a three-week period in August 2014. We advertised to Facebook users who “liked” certain tobacco- or marijuana-related pages (e.g., major cigarette brands and magazines focusing on marijuana) or had identified related interests (e.g., “legalize marijuana”). Advertisements included images of tobacco products, marijuana-related images, and other benign images intended to recruit nonusers. Our recruitment was modeled after other published research methods [14].

### 2.2. Participants

Individuals who clicked on the advertisement were directed to a page describing the survey and the consent statement. Consenting individuals were screened for eligibility (i.e., age); those eligible were forwarded to the online survey, administered via http://www.surveygizmo.com/. To limit duplicate responses, one response per IP address was permitted. The survey took approximately 30 minutes to complete. Participants were compensated $5.

Of the 4510 individuals who started the survey, 2251 did not complete the entire survey (52.6% of whom did not move past the information and consent); 699 were disqualified, including 482 who were outside the age range, 77 who declined consent, and 140 who provided invalid responses. Thus, 1,567 had complete and valid responses. In our sample, 648 (41.6%) were current marijuana users. The average number of days of use in the past 30 days was 17.86 (SD = 11.29; not shown in Table 1). This high prevalence of marijuana use in this sample was intentional, per our recruitment approach targeting tobacco and marijuana users. We intended to ensure that the phenomenon of interest was relevant to the sample, particularly given that the vast majority of the US population does not face issues related to marijuana use in their home [1]. Marijuana users implicitly make decisions about use in the home, and tobacco users are more likely to affiliate with marijuana users [15], suggesting that they may also face decisions about marijuana use in their home. This sample allowed us to explore this phenomenon within a sample where this topic was relevant, including both marijuana users and marijuana nonusers.

### 2.3. Measures

The survey assessed standard sociodemographic factors and health-related factors; health-related factors included in the current analyses are detailed below.

#### 2.3.1. Marijuana Use

Participants were asked, “In the past 30 days, on how many days did you use marijuana (pot, weed, hashish, hash oil)?” [1]. Those using marijuana in the past 30 days were considered current users.

#### 2.3.2. Social Influence

Participants were asked if any parental figures, anyone living in their home, or their significant other uses marijuana and, out of his/her five closest friends, the number who use marijuana [15].

#### 2.3.3. Perceptions of Marijuana

Participants were asked to rate on a scale of 1 = “not at all” to 7 = “extremely” the extent to which they perceived marijuana to be socially acceptable among peers, harmful to health, addictive, and harmful to those exposed to its byproducts. These measures were adapted from prior research [15].

#### 2.3.4. Household Marijuana Rules (HMR)

Participants were asked about rules about marijuana use in their current household. They indicated whether the statements listed in Table 2 were “never true” for them, “sometimes true,” “always true,” or “does not apply.” To quantify the relative restrictions within the sample, an aggregate score of the household marijuana rules (HMR) index was derived by assigning a score of 0 for “never true,” 1 for “sometimes true,” and 2 for “always true” or “does not apply.” “Does not apply” scored 2 because participants reporting this response were not allowing certain behaviors in those contexts by default. In addition, items skipped because of inclusive or restrictive rules leading to a skip pattern were treated as 2's as well. As such, higher scores reflect more restrictions on marijuana in participants' households.

### 2.4. Data Analysis

Participant characteristics were summarized using descriptive statistics. Bivariate analyses were conducted comparing marijuana users versus nonusers and associations with HMR index scores. A multivariable regression model was then developed to identify correlates of HMR, employing backwards stepwise entry at *p* < .10. SPSS 22.0 was used for all data analyses. Statistical significance was set at *α* = .05 for all tests.

## 3. Results

Table 1 presents data indicating that marijuana users versus nonusers were younger (M = 24.49 ± 5.09 versus M = 25.67 ± 5.03, *p* < .001), were less educated (>HS = 63.0% versus 70.7%, *p* < .001), and were more likely to be married or living with a partner (62.3% versus 54.5%, *p* = .001). In terms of social factors, they were more likely to be living with friends/relatives (26.9% versus 20.9%), have parental figures who use marijuana (29.9% versus 8.3%, *p* < .001), have people who live with them use marijuana (48.0% versus 14.0%, *p* < .001), and have a partner who uses marijuana (36.1% versus 6.5%, *p* < .001) but less likely to have children (23.9% versus 32.0%, *p* < .001). Users also had more friends who used marijuana (M = 3.85 ± 1.36 versus M = 1.42 ± 1.55, *p* < .001). In terms of other attitudes about marijuana use, marijuana users versus nonusers perceived marijuana to be more socially acceptable (M = 6.11 ± 1.48 versus M = 4.34 ± 2.26, *p* < .001), less harmful (M = 2.18 ± 1.49 versus M = 3.57 ± 2.42, *p* < .001), and less addictive (M = 2.54 ± 1.79 versus M = 3.40 ± 2.34, *p* < .001) and perceived exposure to marijuana byproducts to be less harmful (M = 2.30 ± 1.66 versus M = 3.75 ± 2.28, *p* < .001).

Overall, 46.0% (24.8% of users; 61.1% of nonusers) reported that marijuana of any type is not allowed on their property (Table 2). In addition to those not allowing it on the property, 6.4% (5.5% of users, 7.7% of nonusers) prohibited marijuana* use* on their property. Of those that allowed some level of marijuana use on their property, 29.2% prohibited marijuana smoking in their home, 11.0% prohibited vaping, eating, or drinking marijuana, and 8.4% prohibited its use in outdoor areas. Only 1.9% of all participants (*N* = 30/1560) allowed marijuana use in the presence of children, with little variability depending on how it was used (e.g., smoked or vaped).

Marijuana users versus nonusers had higher HMR index scores (M = 24.54 ± 8.15 versus M = 29.29 ± 5.59, resp., *p* < .001). Other factors associated with higher HMR scores (Table 1) included younger age (*p* = .006); being female (*p* = .029); lower parental educational background (*p* = .002); being heterosexual (*p* = .035); living with parents or on campus versus other situations (*p* = .001); not having parents, others in the home, or a partner who uses marijuana (*p*'s <.001); having fewer friends who use (*p* < .001); perceiving marijuana use to be less socially acceptable, more harmful, and more addictive (*p*'s <.001); and perceiving marijuana byproducts to be more harmful (*p*'s <.001).

Prior to constructing the multivariable model predicting HMR index scores, we explored collinearity among the predictors of interest. We found that number of friends who smoke was collinear with perceived social acceptability and personal use and that perceptions of harm of marijuana byproducts was collinear with perceived harm of marijuana. Thus, we excluded these two variables and entered all other factors associated with HMR index scores at the *p* < .10. Predictors of higher HMR index scores included younger age (Beta = −0.16, CI −0.22, −0.09), being female (Beta = −0.69, CI −1.31, −0.07), having a Bachelor's degree or higher education (Beta = −0.57, CI −1.06, −0.08), not having a parent (Beta = −2.00, CI −2.88, −1.11) or people living in their home who used/uses marijuana (Beta = −3.40, CI −4.18, −2.63), perceiving marijuana use to be less socially acceptable (Beta = −0.43, CI −0.60, −0.26) and more harmful (Beta = 0.31, CI 0.14, 0.47), and less likelihood of being a marijuana user (Beta = −2.16, CI −2.94, −1.39; adjusted *R* ^2^ = 0.206).

## 4. Discussion

This study is the first to characterize voluntary restrictions on marijuana in home environments. As expected, the newly developed index correlated with theoretical factors per the TPB [11], such as attitudes toward marijuana (e.g., perceptions of harm and addictiveness) and social factors (e.g., important others' marijuana use); these findings are in line with those derived from the home smoking restrictions literature [12, 13]. There was an association between more restrictions and sociodemographics including younger age, gender, and education, which warrant further examination.

In this sample of young adults, nearly half (46.0%) prohibited marijuana on their property, the majority of whom were nonusers. Additionally, a quarter of users prohibited marijuana on their property, implying use outside of the home. There were also some differences in the restriction of smoked marijuana versus noncombustible marijuana in the home, which might suggest that some young adults feel that covert (i.e., noncombustible) use is more acceptable. In addition, a small percentage had rules about use in outdoor areas, which might impact neighbors, be observable by individuals off the property (i.e., on sidewalks), and be subject to legal restrictions, particularly in multiunit housing. Finally, marijuana use in the presence of children was rarely allowed. This is favorable, as substance use by parents and other adults may normalize use and encourage youth initiation.

These findings have implications for research and practice. This index might inform research regarding risks related to marijuana in residential environments, which is critical in a rapidly evolving context of marijuana regulations [10] and the need to intervene to circumvent the potential harms of marijuana secondhand smoke [8] and the impact on youth initiation [9]. Future research should also qualitatively examine how rules are voluntarily adopted and communicated within one's social system and identify other possible rules used in these and other settings. It would also be useful to determine how well individuals understand legal restrictions on use in residential location and whether the rules as they express them are consistent with these laws.

Furthermore, more research is needed to inform the appropriate public health outcome, particularly given the limited research regarding the impact of secondhand marijuana smoke exposure. Specifically, should the objective be a full household ban? If so, where should use be allowed, since use in public is precluded by legalization policies and may have other public health consequences? Alternatively, is the objective to ban smoked forms of marijuana in the home or prevent use in the presence of youth? If so, should noncombustible marijuana use and/or proper storage be encouraged? Even in a time of uncertainty regarding desired outcomes, surveillance regarding how people approach household marijuana rules can inform future efforts to address marijuana use in the home.

## 5. Limitations

Limitations include limited generalizability given that the sample was focused on young adults and specifically targeted marijuana and tobacco users in order to ensure that the phenomenon of interest was relevant to the sample obtained. Also, this sample was mainly drawn from North America and thus is unlikely to represent the attitudes and values of people worldwide. We also had a relatively low response rate, which is open to selection bias. Future research should examine these and other related phenomena among a more representative national sample. Specifically, research might explore other dimensions of how people regulate marijuana use in other settings (e.g., cars). Additionally, the online survey format does not allow us to explore the reasons why individuals might have reported “does not apply” for certain items. Finally, the cross-sectional nature of this study limits the ability to make causal attributions.

## 6. Conclusions

Attitudes about marijuana use and subjective norms related to use are important correlates of allowing marijuana and its use in residential settings. Marijuana users versus nonusers had fewer household marijuana rules. Moreover, use in the presence of children was rare. These findings have implications for future research aimed at objectively examining areas of risk regarding household marijuana rules and informing intervention efforts aimed at reducing exposure to marijuana byproducts and youth exposure to marijuana use.

## Figures and Tables

**Table 1:** Participant characteristics and bivariate analyses demonstrating associations with HMR index scores.

Variable	Total *N* = 1567 *N* (%) or M (SD)	Association with HMR M (SD) or *r*	*p* value
*Sociodemographics*			
Age (SD)	25.18 (5.09)	−0.07	.006
Gender (%)			.029
Male	766 (49.1)	26.94 (7.41)	
Female	776 (49.7)	27.73 (6.89)	
Race (%)			.151
White	1356 (86.9)	27.22 (7.22)	
Other	204 (13.1)	28.00 (6.72)	
Ethnicity (%)			.785
Hispanic/Latino	201 (13.0)	27.44 (7.23)	
Other	1341 (87.0)	27.29 (7.17)	
Education (%)			.083
≤High school	508 (32.6)	27.89 (6.99)	
Some college	795 (51.0)	26.99 (7.34)	
≥Bachelor's degree	257 (16.5)	27.24 (6.90)	
Parental education (%)			.002
≤High school	437 (28.0)	28.34 (6.68)	
Some college	489 (31.4)	27.04 (7.14)	
≥Bachelor's degree	633 (40.6)	26.83 (7.44)	
Employment status (%)			.393
Employed full or part time	788 (50.5)	27.11 (7.34)	
Full or part time student	334 (21.4)	27.36 (6.78)	
Unemployed/other	438 (28.1)	27.69 (7.11)	
Sexual orientation (%)			.035
Heterosexual	1222 (78.6)	27.53 (7.00)	
Other	333 (21.4)	26.59 (7.68)	
Community type (%)			.133
Rural	400 (25.6)	27.68 (6.82)	
Urban	491 (31.5)	26.80 (7.49)	
Suburban	669 (42.9)	27.49 (7.10)	
*Social factors*			
Relationship status (%)			.748
Married/living with partner	659 (42.3)	27.27 (7.17)	
Other	900 (57.7)	27.83 (7.15)	
Living situation (%)			.001
Live alone	133 (8.5)	27.29 (6.68)	
Live with spouse/partner	600 (38.5)	27.14 (7.16)	
Live with friends/relatives	364 (23.3)	26.13 (8.07)	
Live with parents	347 (22.2)	28.47 (6.47)	
Live on campus	81 (5.2)	28.64 (6.31)	
Other	35 (2.2)	28.69 (4.99)	
Have children (%)			.426
No	1112 (71.3)	27.23 (7.22)	
Yes	448 (28.7)	27.55 (7.02)	
Parental figure uses marijuana			<.001
No	1290 (82.7)	28.02 (6.64)	
Yes	270 (17.3)	23.99 (8.51)	
People who live with you use marijuana			<.001
No	1121 (71.9)	28.82 (6.10)	
Yes	270 (17.3)	23.51 (8.20)	
Partner uses marijuana			<.001
No partner/no use	1265 (81.1)	28.15 (6.52)	
Yes	294 (18.9)	23.76 (8.60)	
Number of 5 closest friends using marijuana	2.43 (1.90)	−0.34	<.001
Social acceptability of marijuana use	5.08 (2.15)	−0.28	<.001
*Perceptions of harm and use*			
Harm to health of marijuana use	2.99 (2.08)	0.22	<.001
Harm to health of marijuana byproducts	3.14 (2.16)	0.22	<.001
Addictiveness of marijuana	3.04 (2.17)	0.10	<.001

**Table 2:** Household marijuana rules (HMR) index items.

Variable	Never true *N* (%)	Sometimes true *N* (%)	Always true *N* (%)	Does not apply *N* (%)
*General rules*				
(1) Marijuana of any type is not allowed in your home or on your property. (*If always true, skip to next section*) (*i.e., after item *(*16*))	379 (24.3)	218 (14.0)	718 (46.0)	245 (24.3)
(2) The *use *of any type of marijuana is not allowed in your home or on your property; that is, marijuana use is not allowed anywhere inside your home or in outdoor areas on your property such as decks, garages, or porches or shared areas with neighbors. (*If always true, skip to the question *(*14*))	338 (56.7)	200 (33.6)	38 (6.4)	20 (3.4)
*Indoor policies*				
(3) Smoking marijuana is not allowed anywhere inside your home.	240 (44.7)	135 (25.1)	157 (29.2)	5 (0.9)
(4) Vaping, eating, or drinking marijuana products is not allowed anywhere inside your home.	332 (61.9)	118 (22.0)	59 (11.0)	27 (5.0)
*Outdoor policies*				
(5) The use of any type of marijuana is not allowed in outdoor areas on your property, such as decks, garages, or porches. (*If always true, skip to the question *(*8*))	320 (59.5)	151 (28.1)	45 (8.4)	22 (4.1)
(6) Smoking marijuana is not allowed in outdoor areas on your property, such as decks, garages, or porches.	291 (63.7)	161 (35.2)	3 (0.7)	2 (0.4)
(7) Vaping, eating, or drinking marijuana products is not allowed in outdoor areas on your property, such as decks, garages, or porches.	328 (70.5)	117 (25.2)	5 (1.1)	15 (3.2)
*Shared or community areas*				
(8) The use of any type of marijuana is not allowed in shared areas with neighbors, such as hallways, lobbies, or courtyards. (Note: If you do not share areas with neighbors, select “does not apply.” *If always true or does not apply, skip to the question *(*11*))	60 (11.2)	62 (11.6)	134 (25.0)	279 (52.1)
(9) Smoking marijuana is not allowed in shared areas with neighbors, such as hallways, lobbies, or courtyards.	47 (40.5)	61 (52.6)	3 (2.6)	5 (4.3)
(10) Vaping, eating, or drinking products marijuana is not allowed in shared areas with neighbors, such as hallways, lobbies, or courtyards.	60 (49.6)	49 (40.5)	4 (3.3)	8 (6.6)
*When children are present*				
(11) The use of any type of marijuana is not allowed in your home (indoors and outdoors) when children are present. (*If always true, skip to question *(*14*))	30 (5.6)	73 (13.6)	372 (69.5)	60 (11.2)
(12) Smoking marijuana is not allowed in your home when children are present.	22 (22.0)	56 (56.0)	22 (22.0)	—
(13) Vaping, eating, or drinking marijuana products is not allowed in your home when children are present.	38 (37.6)	43 (42.6)	13 (12.9)	7 (6.9)
*Growing and storage*				
(14) The growing of marijuana on your property is not allowed.	69 (11.9)	55 (9.5)	395 (67.9)	63 (10.8)
(15) The storage of marijuana in your home is not allowed. (*If always true, skip to the next section *(*i.e., after item *(*16*)))	249 (42.1)	121 (20.4)	197 (33.3)	25 (4.2)
(16) In your home, marijuana is stored in locked containers/areas or in a location out of the reach of children.	23 (9.1)	23 (9.1)	176 (69.3)	32 (12.6)
